# From the Editor-in-Chief

**DOI:** 10.2188/jea.JE20090193

**Published:** 2010-03-05

**Authors:** Tomotaka Sobue

Two years have passed since I assumed the position of Editor-in-Chief (EIC). When I began, my predecessor had done a great deal of superlative work for the journal, indeed so much that it seemed almost impossible for me to advance the Journal further. I decided to focus my efforts on establishing an editorial system that would not be dependent on the talents of any particular EIC.

In October 2008, the editorial board introduced an online submission system (Manuscript Central^®^). In addition, all accepted manuscripts are now systematically copyedited. Finally, the editorial board, which consists of the 18 associate editors and the production manager, now meets regularly. These changes allow the Journal to function somewhat more independently of the EIC, and ensure continuity when the Editor changes. Of course, the success of this system owes much to the considerable efforts of all the members of the editorial board.

To further develop and improve our editorial system, we need to balance the need for high-quality research articles with our desire to offer the chance of publication to authors working in varying research contexts around the world, and it is my belief that article acceptance should not depend solely on the application of strict criteria for publication. However, whatever the system used to evaluate submissions, the merit of our Journal absolutely depends on the depth and quality of the comments made by reviewers, most of whom are members of the Japan Epidemiological Association. The editorial board gratefully acknowledges their efforts and looks forward to further contributions from them.

The editorial board recognizes that each submitted article is the fruit of authors’ painstaking research, and we maintain the greatest respect for them throughout the editorial process. At the same time, the Journal is aware of the risks of scholarly misconduct, which include fabrication, falsification, and plagiarism. We have therefore decided to introduce the CrossCheck software program—which compares submitted manuscripts with those already published—to identify instances of plagiarism. This system is not foolproof, however, so we will continue to improve editorial oversight using our human and technological resources.

Tomotaka Sobue, MD, MPH

Editor-in-Chief, Journal of Epidemiology

